# Identification of a novel frameshift variation in ANKRD11: a case report of KBG syndrome

**DOI:** 10.3389/fgene.2024.1439905

**Published:** 2025-01-03

**Authors:** Qing Shao, Qiang Jiang, Yuqi Luo, Yimei Meng, Guoyu Tian, Xiao Yin

**Affiliations:** Department of Endocrinology and Metabolic Diseases, Shandong First University Affiliated Central Hospital, Jinan, China

**Keywords:** KBG syndrome, ANKRD11, frameshift variation, noonansyndrome, RRAS2

## Abstract

**Background:**

KBG syndrome (KBGS, OMIM: 148050) is a rare genetic disorder characterized by macrodontia, short stature, skeletal abnormalities, and neurological manifestations. The objective of this study is to investigate a case of KBG syndrome caused by a novel frameshift mutation in ANKRD11.

**Methods and results:**

We present the case of an 18-year-old Chinese male exhibiting characteristic features including a triangular face, micrognathia, hypertelorism, macrodontia, bushy eyebrows, prominent ears, short stature, low hairline, delayed cognitive development, and scoliosis. Whole exome sequencing identified a novel frameshift variant in the ANKRD11 gene which ultimately led to the diagnosis of KBG syndrome.

**Conclusion:**

In this study we have identified a previously unreported frameshift variant (NM_013275.6:c.2589dup) in ANKRD11 that causes KBG syndrome. This finding expands both the molecular and clinical spectrum of this rare genetic disease.

## Introduction

KBG syndrome is a rare autosomal dominant genetic disorder characterized by enlarged upper central incisor teeth, distinctive craniofacial features (such as a triangular face, prominent nose bridge, thin upper lip), skeletal manifestations (including short stature, delayed bone age, various rib and vertebral abnormalities), and intellectual disability. The phenotypic spectrum of KBG syndrome is highly diverse. According to the literature, macrodontia of the permanent upper incisors is reported in 85%–95% of individuals with KBG syndrome, making it the most prevalent dental characteristic. Hearing impairment has been observed in approximately 25%–31% of patients. Postnatal short stature is a common feature among individuals with KBG syndrome, and there have been promising reports regarding the response to growth hormone therapy (Ho et al., 2022). Nonspecific or mild symptoms often go underdiagnosed or unnoticed. The prevalence of KBG syndrome does not differ across ethnic groups, and although it follows an autosomal dominant inheritance pattern, it occurs more frequently in males than females for reasons that remain unknown (Choi et al., 2022). It is caused by dominant variants of the ANKRD11 gene or microdeletions of 16q24.3 containing the ANKRD11 gene (Martinez-Cayuelas et al., 2022; Niihori et al., 2019). The ANKRD11 gene is a gene located on chromosome 16q24.3 and comprises 11 exons. Functionally, ANKRD11 serves as a crucial co-regulator that recruits chromatin remodelers to target genes through specific interactions with transcriptional repressors or activators, thereby modulating gene expression (Parenti et al., 2021).

Here we report an 18-year-old Chinese male with ANKRD11 (NM_013275.6:c.2589dup) frameshift mutation detected by Sanger sequencing. Combined with clinical features and family mapping analysis, we conclusively diagnosed the patient with KBG syndrome.

## Case report and methodologies

### Case report

This case presents the clinical report of an 18-year-old male patient who was initially admitted to Jinan Central Hospital in Shandong Province on 2 November 2023 and subsequently transferred to our department on 11 November 2023. He is the second child born via cesarean section and there is no consanguinity between his parents. The patient had a birth weight of 3.35 kg and underwent surgical correction for undescended testicles at approximately 2 years of age. Additionally, he received a frenectomy procedure for lingual frenulum release. Since birth, he has exhibited growth retardation which went unnoticed by family members without any intervention. His stature lags behind that of his peers and he experiences delayed cognitive development, introverted personality traits, and speech fluency issues. Over the past year, he has reported chest pain along with incomplete eye closure during sleep at night accompanied by frequent nightmares and episodes of vocalization.

**FIGURE 1:**
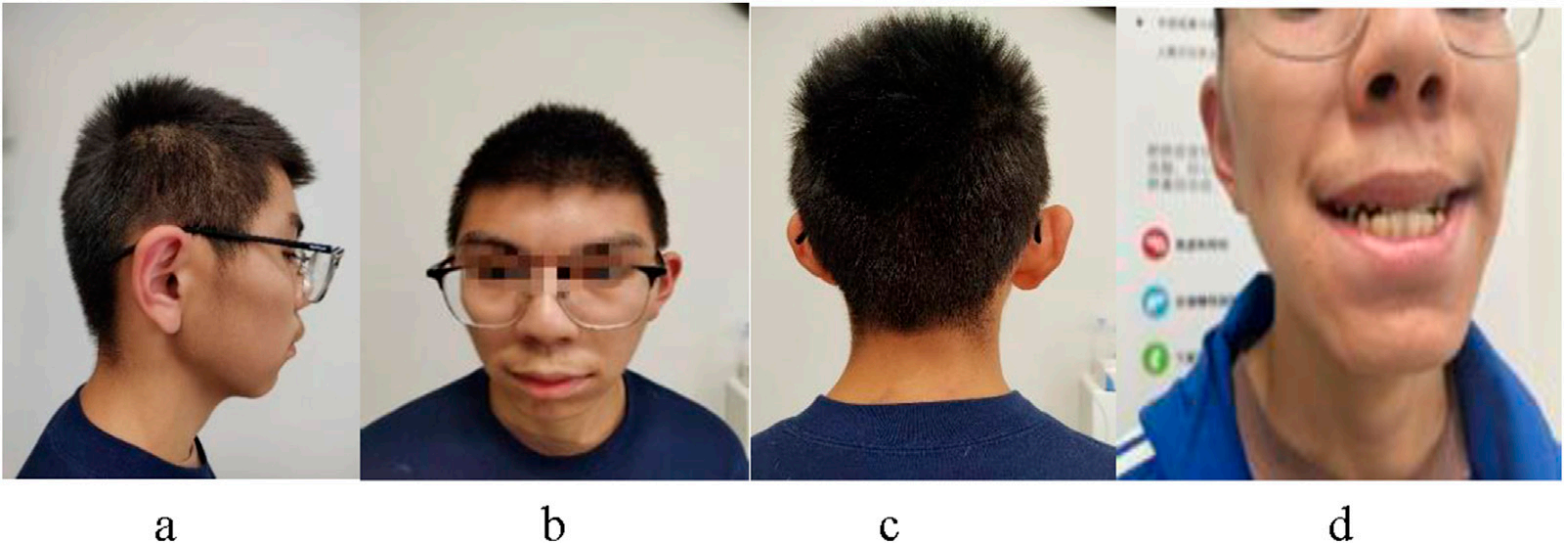
The patient presents with a characteristic facial profile, characterized by protruding ears **(A)**, a triangular face shape, widely spaced eyes, broad or bushy eyebrows, nose bridge **(B)**, hair abnormalities **(C)**, and prominent upper central incisor teeth **(D)**.

The physical examination revealed distinctive facial features, including a triangular face shape, small jaw size, wide interocular distance, large incisor teeth, prominent eyebrows, and medium-length protruding ears (Figure 1). The height measured during the examination was recorded as 162.5 cm, while the weight was noted as 50.7 kg with a waist circumference of 65 cm. Additionally, the whole arm length measured 164 cm; upper arm length measured 70 cm; leg length measured 97 cm. There was right curvature in the thoracic vertebrae, secondary sexual characteristics were observed to have developed normally.

### Methodologies

According to the standardized growth curve of height in Chinese children and adolescents aged 0–18 years, his height falls within a range that is 1-2 standard deviations below the average height for children of the same sex, age, and race. The subject’s hormone levels were as follows: prolactin at 16.4 ng/mL (reference range: 2.52–13.23 ng/mL) and estradiol at 78.2 pmol/L (reference range: 88.8–227.9 pmol/L). Other parameters include glucose tolerance, insulin-like growth factor 1, serum growth hormone, insulin-like growth factor binding protein-3, thyroid function, parathyroid hormone, carboxyl terminal propeptide (pICp) of type I collagen, 25 hydroxyvitamin D, β-collagen degradation products and serum osteocalcin were within normal ranges. No evidence of growth hormone deficiency was observed during the arginine stimulation test.

**FIGURE 2:**
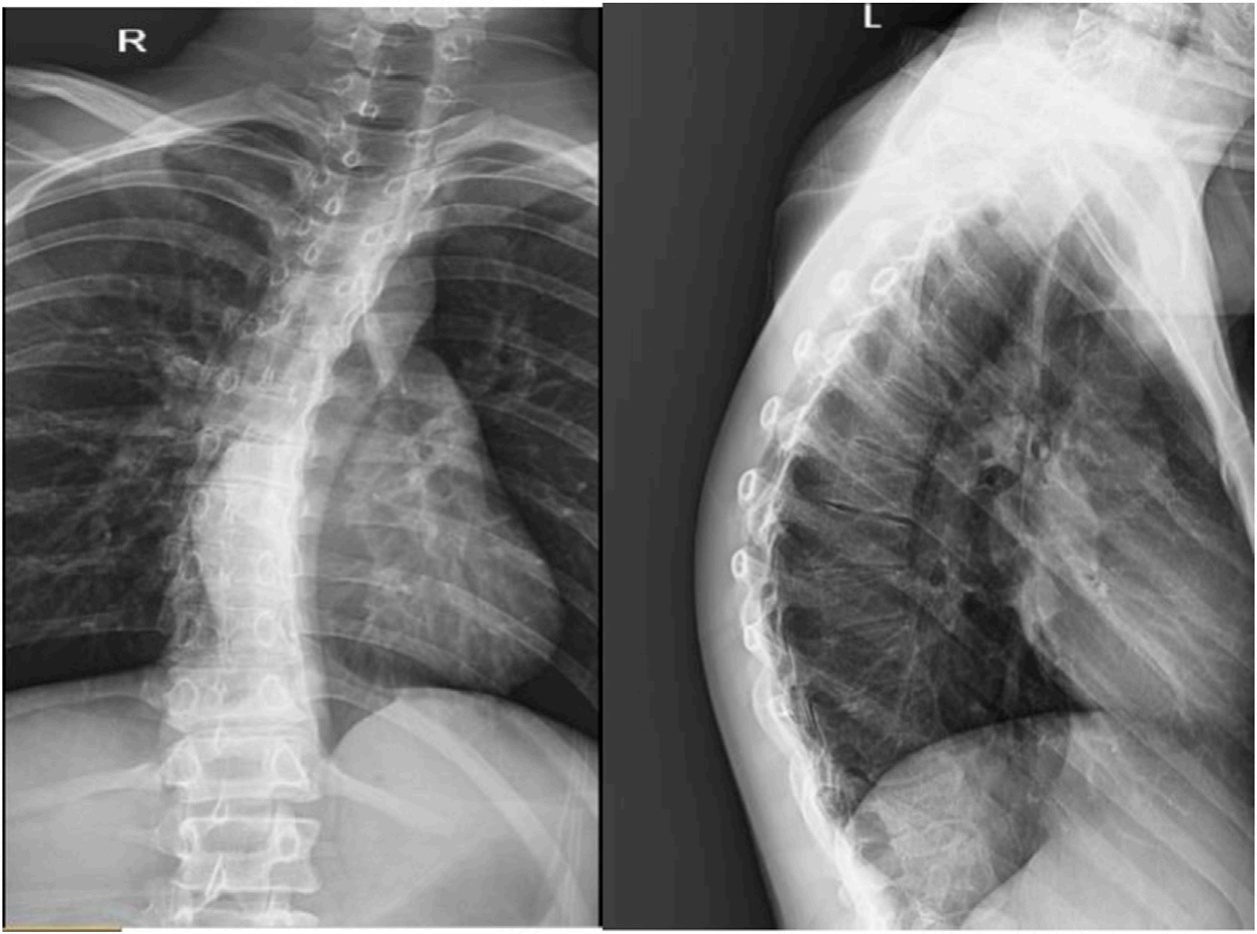
Anteroposterior and lateral view of the patient’s spine: The thoracic spine exhibited scoliosis with a localized posterior protrusion.

**FIGURE 3:**
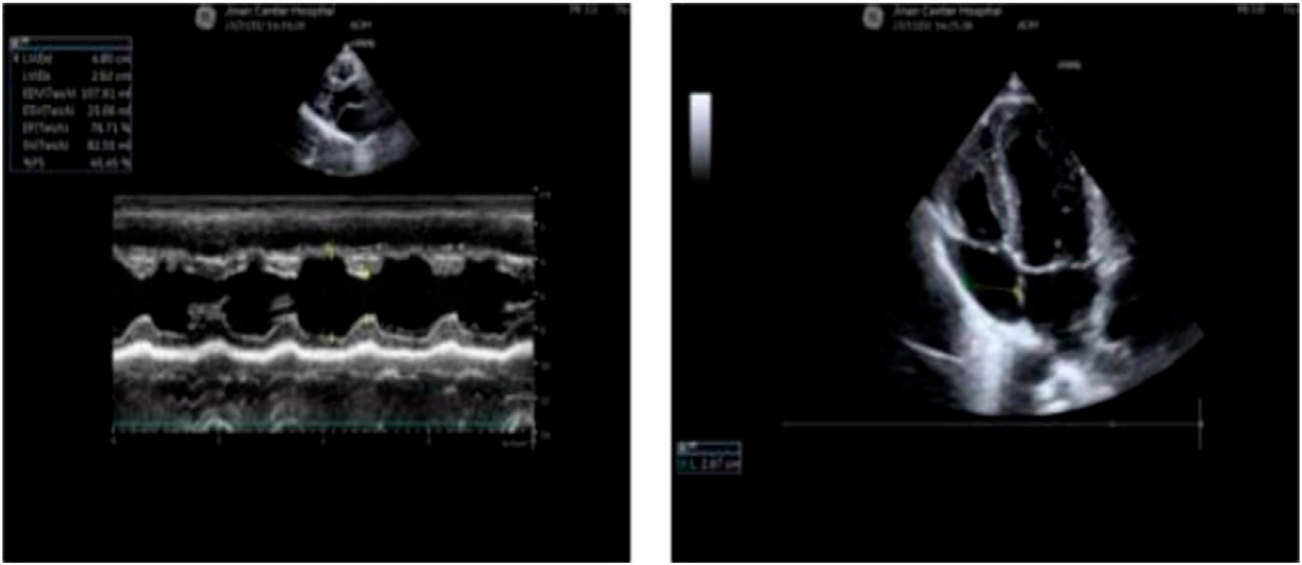
The patient’s cardiac ultrasound revealed the presence of mild tricuspid regurgitation.

**FIGURE 4:**
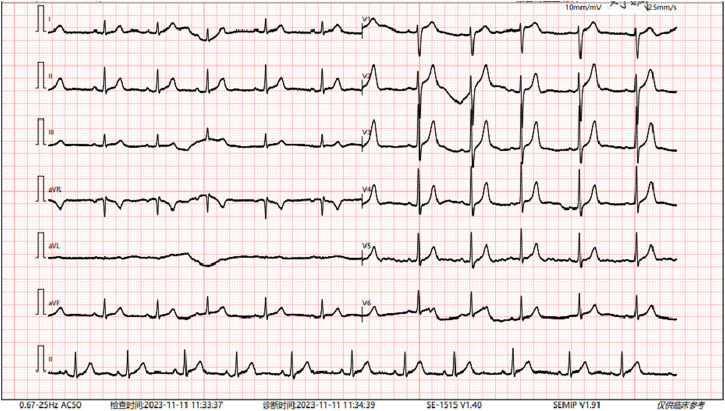
The patient’s electrocardiogram indicates sinus arrhythmia.

Radiographs revealed a hyperflexion position with lateral curvature of the thoracic vertebrae, accompanied by a localized posterior process of the thoracic vertebrae (Figure 2). Cardiac ultrasound demonstrated mild tricuspid valve regurgitation (Figure 3), while ECG indicated sinus arrhythmia (Figure 4).

Initially considering Noonan syndrome as a potential genetic condition for this patient’s symptoms, whole exome sequencing was conducted on both the patient and his parents to further investigate possible mutations (Tables 1, 2). With the informed consent of the patients and their parents, a peripheral blood sample of 5 mL was collected from each participant. The blood samples were then subjected to EDTA anticoagulation treatment before being utilized for subsequent molecular testing. Disease-related genes were analyzed through whole exome sequencing at Guangzhou Jin Yu Medical Laboratory Company. Genomic DNA was extracted from the samples and underwent fragmentation, splicing, amplification, and purification processes to construct a DNA library using hybrid capture technology. The NovaSeq 6000 high-throughput sequencing platform was employed to examine both exon regions and lateral intron regions of all 20,099 human genome genes.

**TABLE 1:** A novel frameshift mutation in ANKRD11 gene was identified.

Gene	Chromosome location	Variation information	Zygote type	Disease name	Genetic pattern	Variation source	Variation classification
ANKRD11	chr16: 89350361	NM_013275.6:c.2589dup (p.Asp864Argfs*52)	Heterozygous	KBG syndrome [MIM:148050]	AD	Emerging variation	Pathogenic variation

**TABLE 2:** Missense variants in the RRAS2 gene were inherited from his father.

Gene	Chromosome location	Variation information	Zygote type	Disease name	Genetic pattern	Variation source	Variation classification
RRAS2	chr11:14317323	NM 02250.6:c.187C>T (p.Arg63Trp)	Heterozygous	NoonanSyndrome type12 [MIM:618624]	AD	father	Clinical significance is unknown

A frameshift mutation c.2589dup (p.Asp864Argfs*52) was identified in the ANKRD11 gene due to non-triplet base repeats, which could theoretically lead to nonsense-mediated mRNA degradation or early termination of coding amino acid sequence results in loss of normal protein function.

Sanger sequencing confirmed the absence of this mutation in both parent’s samples, suggesting it is a *de novo* mutation. Based on available evidence, this variant is classified as pathogenic since pathogenic mutations in the ANKRD11 gene are known to cause KBG syndrome.

The subject also carries a missense variation, c.187C>T (p.Arg63Trp), in the coding region of the RRAS2 gene, which was confirmed by Sanger sequencing to be inherited from the father. Pathogenic variations in the RRAS2 gene have been associated with Noonan Syndrome type 12. Although this variant corresponds to the subject’s primary clinical phenotype, its clinical significance is currently unknown or does not match the expected genetic pattern of the disease.

The KBG syndrome is a rare autosomal dominant genetic disorder caused by dominant variants of the ANKRD11 gene or microdeletions of 16q24.3 containing the ANKRD11 gene. According to the 2016 KBG revision diagnosis (Low et al., 2016), clinical diagnosis can be established if two major criteria or one major criterion and two minor criteria are met: Primary criteria include significant developmental delays or mild/moderate intellectual disabilities or learning difficulties, characteristic facial features such as a triangular face, short head, widely spaced eyes, broad or bushy eyebrows, protruding ears and nose bridge, bulbous nose with forward-leaning nostrils, medium-long thin vermilion upper lip; postpartum microsomia; and having a first-degree relative with KBG syndrome. Secondary criteria consist of conductive hearing loss due to recurrent otitis media, palatal anomalies, hair abnormalities (e.g., low hairline and coarse hair), delayed bone age (2 standard deviations below average), large anterior fontanelle with delayed closure, abnormal hand structure costovertebral anomalies scoliosis electroencephalogram abnormalities with or without seizures dysphoria male cryptorchidism. The patient exhibits a typical facial profile along with huge upper central incisor teeth, hair abnormalities, costovertebral anomaly, and undescended testicles, while meeting three primary criteria and three secondary criteria, KBG syndrome can be clinically diagnosed. In conjunction with the pathogenic mutation of the ANKRD11 gene, the patient was diagnosed with KBG syndrome.

## Discussion

KBG syndrome (OMIM# 148050) is a rare autosomal dominant hereditary disorder, initially documented by Hermann et al., in 1975. It is characterized by prominent clinical features including macrodontia (particularly of the upper and middle incisors), distinctive facial characteristics (triangular face, brachycephaly, hypertelorism, wide interocular distance, broad or thick eyebrows, prominent ears, prominent nasal bridge, bulbous nose with anteverted nostrils, thin vermilion upper lip), short stature, developmental delay/intellectual disability as well as behavioral issues (Martinez-Cayuelas et al., 2022). Affected individuals may also experience feeding difficulties during infancy, skeletal anomalies such as brachydactyly delayed closure of the anterior fontanelle scoliosis. Additionally observed are hearing impairments encompassing conductive/mixed/sensorineural types of loss alongside seizure disorders and brain malformations.

The causative gene for this syndrome, ANKRD11, was identified in 2011 by Sirmaci et al. (2011). To date, a total of 1181 gene mutations of ANKRD11 have been cataloged in the NCBI (National Center for Biotechnology Information) database, with 590 attributed to KBG syndrome. Notably, there is no record of the ANKRD11 (NM_013275.5:c.2589dup) frameshift variant among the identified variants of the ANKRD11 gene associated with KBG syndrome in the NCBI database to date. A study conducted in 2020 involving 13 Chinese patients with KBG syndrome revealed that truncating variants in the ANKRD11 gene were more likely to be associated with global growth retardation and intellectual disability/learning difficulties compared to missense variants in the same gene (Gao et al., 2022). ANKRD11 functions as a co-regulator during the process of brain development and plays a pivotal role in regulating the proliferation of neural progenitor cells, as well as in facilitating the generation and precise localization of newborn neurons, neuronal plasticity, and dendritic differentiation (Amllal et al., 2023; Zhang et al., 2023).

RRAS2, a member of the RAS GTPases superfamily, regulates multiple cellular processes such as proliferation, survival, and migration. Dysregulation of RRAS2 has been associated with tumorigenesis (Capri et al., 2019). The missense variation of RRAS2 in this patient suggests the possibility of Noonan syndrome type 12. Noonan syndrome (NS) is characterized by distinctive facial features, including hypertelorism, epicanthus, ptosis and slanting eyelids, thick auricles, low-set and supination ears with prominent shape. It is also associated with short stature, congenital heart defects, and varying degrees of developmental delay. Additional findings may include a wide or webbed neck, abnormal chest shape (upper convex thorax and lower concave thorax), cryptorchidism, various coagulation defects, lymphatic dysplasia, and ocular abnormalities. Most cases of Noonan syndrome are inherited in an autosomal dominant manner; however, among the known variants, only those caused by LZTR1 pathogenic variants can be inherited as either autosomal dominant or autosomal recessive (Roberts, 2001).

In 2019, a study was conducted on patients from six families who exhibited typical features of Noonan syndrome but did not have gene mutations associated with previously known cases (Niihori et al., 2019). Consequently, the study identified a novel gene mutation, RRAS2, which is a rare autosomal dominant inheritance mutation in Noonan syndrome. The specific mutations found were c.70_78dup (p.Gly24_Gly26dup), c.[216A > T; 224T > G] (p.[Gln72His; Phe75Cys]), c.215A > T (p.Gln72Leu), c.65_73dup (p.Gly22_Gly24dup), c.68G > T (p.Gly23Val), and c.208G > A (p.Ala70Thr) (Yu et al., 2023). However, it should be noted that NS caused by the RRAS2 mutation follows an autosomal dominant inheritance pattern. In this particular case, the patient inherited the RRAS2 mutation from his healthy father. No variations of the RRAS2 gene [02250.6:c187C>T(p.Arg63Trp)] have been reported in association with Noonan syndrome.

Combined with clinical features and family mapping analysis, we conclusively diagnosed the patient with KBG syndrome. The possibility of Noonan syndrome is unlikely in this case. Prenatal diagnosis serves as an effective measure to prevent disease recurrence within the family for individuals with KBG syndrome. The reported case we present contributes to the expansion of the genetic variant spectrum associated with KBG syndrome. This previously unreported gene mutation not only enhances our molecular understanding broadens the clinical spectrum of this rare genetic disorder, providing valuable insights for medical professionals studying this condition.

## Data Availability

The datasets presented in this study can be found in online repositories. The names of the repository/repositories and accession number(s) can be found in the article/supplementary material.
